# Modeling the process of childhood *ETV6-RUNX1* B-cell leukemias

**DOI:** 10.18632/oncotarget.21281

**Published:** 2017-09-27

**Authors:** Guillermo Rodríguez-Hernández, Daniel Schäfer, Ana Gavilán, Carolina Vicente-Dueñas, Julia Hauer, Arndt Borkhardt, Isidro Sánchez-García

**Affiliations:** ^1^ Experimental Therapeutics and Translational Oncology Program, Instituto de Biología Molecular y Celular del Cáncer, CSIC/ Universidad de Salamanca, Campus M. de Unamuno s/n, Salamanca, Spain; ^2^ Institute of Biomedical Research of Salamanca (IBSAL), Salamanca, Spain; ^3^ Department of Pediatric Oncology, Hematology and Clinical Immunology, Heinrich-Heine University Düsseldorf, Medical Faculty, Düsseldorf, Germany

**Keywords:** epigenetic modulation, childhood leukemia, mutational pattern, GEMM, infection exposure

## Abstract

*ETV6-RUNX1* is associated with the most common subtype of childhood leukemia. Pre-leukaemic clones carrying *ETV6-RUNX1* oncogenic lesions are frequently found in neonatal cord blood, but only few *ETV6-RUNX1* carriers develop pB-ALL. The highly demanding and pending challenge is to reveal the multistep natural history of *ETV6-RUNX1* pB-ALL, because it can offer non-toxic prophylactic interventions to preleukemic carriers. However, the lack of a genetically engineered *ETV6-RUNX1* mouse model mimicking the human pB-ALL has hampered our understanding of the pathogenesis of this disease. This rule has now been broken in a study of the effect of the *ETV6-RUNX1* oncogene in cancer development in a mouse model in which oncogene expression is restricted to the stem cell compartment. In this article, we review the different attempts to model this disease, including the recent representative success stories and we discuss its potential application to both identify etiologic factors of childhood *ETV6-RUNX1* pB-ALL and prevent the conversion of a preleukemic clone in an irreversible transformed state.

## THE BIOLOGY OF CHILDHOOD ETV6-RUNX1+ B-ALL

The *ETV6-RUNX1* fusion gene is the most common chromosomal alteration in pediatric cancer and occurs in approximately 25% of childhood B cell precursor-acute lymphoblastic leukemia (pB-ALL) [1]. The t(12;21)(p13;q22) chromosomal translocation results in the fusion of two critical regulators of hematopoiesis, bringing together the 5′ portion of the *ETV6* (*TEL*) gene on chromosome 12p13 and nearly the entire *RUNX1* (*AML1*) gene on chromosome 21q22 [2–4]. The overall cure rate is excellent (approximately 90%), however, treatment is associated with severe toxic side effects, long-term sequelae and even though, 20% of children still relapse and may later succumb to their disease [5]. Preventional strategies are clearly superior to any therapy improvement. The prerequisite to develop these strategies is to discover the aetiology of ETV6-RUNX1 pB-ALL.

The *ETV6-RUNX1* fusion gene is one of the most extreme examples of genotype-phenotype association. Its presence is only associated to pB-ALL. ETV6-RUNX1 pB-ALL is a clonal malignant disease that originates in a single cell and is characterized by an accumulation of immature B-cells that are phenotypically reminiscent of normal stages of B-cell differentiation. This finally leads to the suppression of normal hematopoiesis and the infiltration of many vital organs. The cells in ETV6-RUNX1 pB-ALL are generally regarded as malignant counterparts of normal B-cell precursors, but ETV6-RUNX1 pB-ALL origin is still the subject of continuing discussion, given the fact that human disease is diagnosed at late stages and cannot be monitored during its natural evolution from its cell of origin [6–8].

**Figure 1:**
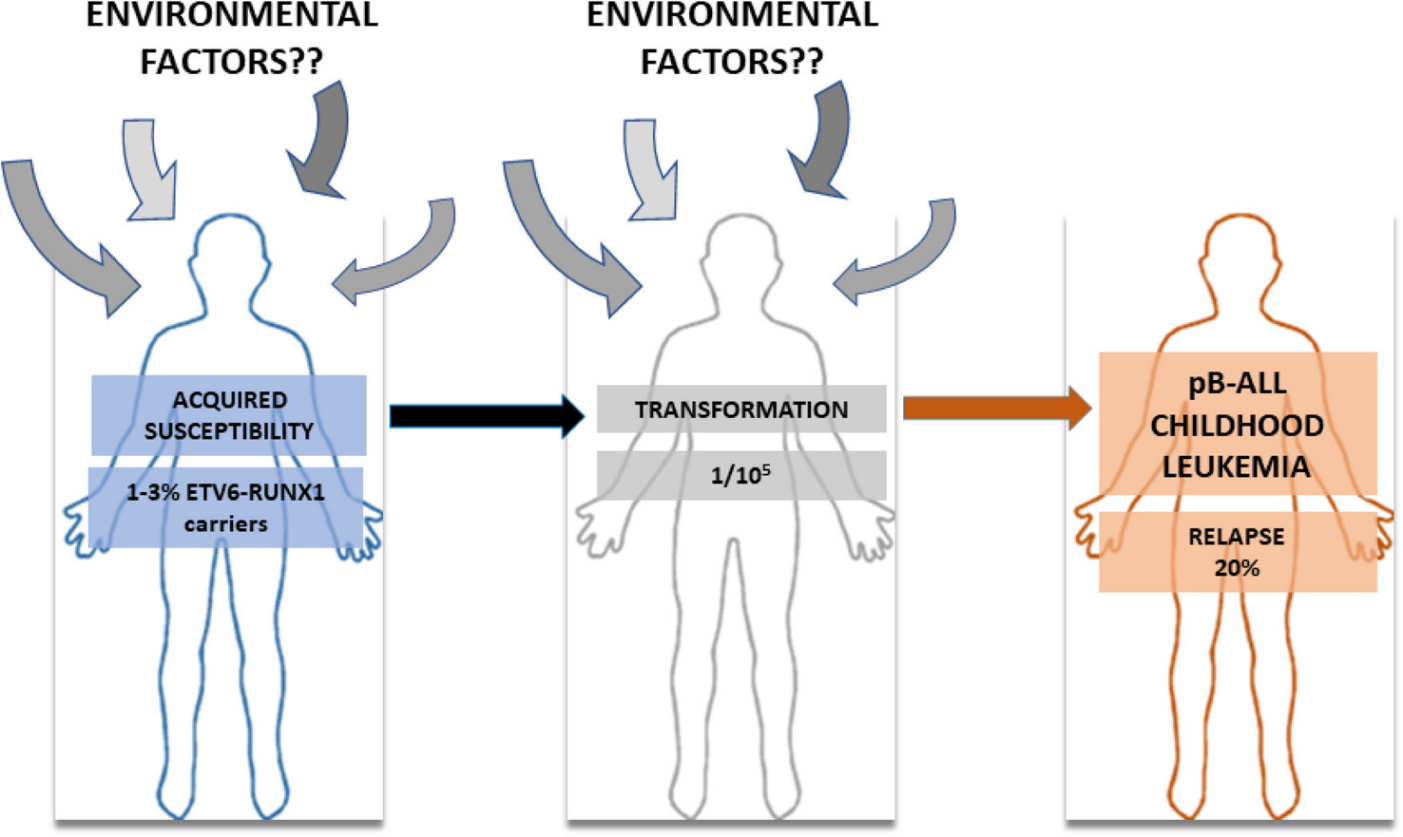
The natural history of ETV6-RUNX1 pB-ALL childhood leukemia Between 1–3% of pre-leukemic clones carrying *ETV6-RUNX1* translocation are frequently found in neonatal cord blood, but only few *ETV6-RUNX1* carries develop pB-ALL (1/10^5^). Many environmental factors have been pointed out as implicated in this transformation process but the lack of biological evidence has kept environmental factor under suspicion.

Pre-leukaemic clones carrying *ETV6-RUNX1* oncogenic lesions are frequently found in neonatal cord blood [9, 10], but only few *ETV6-RUNX1* carriers develop the disease (Figure 1). Thus, *ETV6-RUNX1* gene fusion seems to confer a low risk of developing pB-ALL and represents the first event (“hit”) in the process of leukemogenesis creating a preleukemic clone, which requires secondary postnatal genetic aberrations. A better understanding of both, what causes the initial changes and which events are leading to an irreversibly transformed state can potentially allow to prevent pB-ALL development in preleukemic carriers. Thus, we can now consider childhood leukemia research in terms of seeking to prevent the occurrence of this disease. This conceptual framework requires a precise understanding of the genetic and epigenetic factors that promote pB-ALL development, and how these are influenced by environmental factors associated to human leukemia development such as infection, low-dose radiation, etc (Figure 1). However, the molecular events involved in the early genesis of ETV6-RUNX1-associated leukemia have been particularly elusive because these stages are usually not detected in children and, when they are, very little if any tissue is available for research studies. Therefore, preclinical models of ETV6-RUNX1-associated pB-ALL are an essential unmet need to prevent the occurrence of this disease.

### Zebrafish transgenic models for childhood ETV6-RUNX1+ pB-ALL

Zebrafish has been instrumental to delineate the cell-of-origin where the *ETV6-RUNX1* fusion gene could facilitate the development of pB-ALL. When *ETV6-RUNX1* expression was under the control of the *rag2* promoter, zebrafish transgenic did not develop any leukemia, suggesting that the cell-of-origin of pB-ALL is not a lymphoid committed progenitor [11]. On the contrary, *ETV6-RUNX1* expression under the control of ubiquitous promoters in zebrafish resulted in precursor B-cell malignancies, suggesting that ETV6-RUNX1-associated leukemias only might develop when ETV6-RUNX1 was expressed at the level of non-committed progenitors [11]. Like in humans, these B-cell leukemias appeared with low penetrance and long latency suggesting the need for a second hit. However, this zebrafish transgenic model has not been able to identify the molecular events involved in the early genesis of ETV6-RUNX1-associated leukemia and to define if the environmental factors associated to the human disease influence leukemia disease development in the zebrafish transgenic model.

### Murine mouse models for childhood ETV6-RUNX1+ pB-ALL

Transplantation of ETV6-RUNX1-expressing human or murine B cells into immunodeficient mice has been one of the main approaches used to try to understand childhood ETV6-RUNX1+ pB-ALL development. In agreement with the zebrafish observations mentioned above, initial studies in pro-B cell lines shown that ETV6-RUNX1 by itself cannot confer cytokine factor-independent growth (Table 1) [12]. However, sporadic T- or B-cell leukemias appeared when total bone marrow cells from wild-type mice were transduced with *ETV6-RUNX1*, suggesting that an uncommitted progenitor could the cell-of-origin of ETV6-RUNX1+ leukemia [13]. Similarly, mice reconstituted with transduced hematopoietic progenitors with *ETV6-RUNX1* did not develop leukemia, but a bias in the differentiation of the progenitors towards B cell development was observed [14]. Overall, the transplantation of ETV6-RUNX1-expressing human or murine B cells into immunodeficient mice has failed to reproduce the childhood ETV6-RUNX1+ pB-ALL [15, 16]. In addition, this technological approach presents many disadvantages. First, these models lack an immune system in contrast to children where the leukemia appears. Second, the lack of reproducibility and homogeneity is given by the fact that many parameters (injection site, sorting parameters, pre-treatment of recipients with specific antibodies, etc) influence the grafting efficiency and capacity of the injected cells in the recipient animals [6]. In addition, another main problem is that recipient mice should be irradiated with gamma radiation before the injection of transduced cells, rendering these approaches impractical for the study of the molecular events involved in the early genesis of ETV6-RUNX1-associated leukemia and the contribution of environmental factors associated to the human disease to leukemia development. Consequently, in order to accurately reproduce all the aspects intervening in B-ALL development, it is necessary to perform experiments in an *in vivo* setting in which the leukemia emerges in the appropriate microenvironment.

**Table 1:** Main experimental models of ETV6-RUNX1

Experimental design	Phenotype	Year	Reference
Immunoglobulin heavy chain enhancer/promoter	The mice did not develop leukemia.	2001	[12]
Retroviral gene transfer	T cell leukemia and myeloid leukemia	2002	[13]
Fetal liver HPCs transduced with retroviral vectors	ETV6-RUNX1 did not induce leukemia in transplanted mice.	2004	[14]
Bone marrow transplantation model	No leukemia development was observed.	2005	[15]
ETV6-RUNX1 expression is conditionally driven from the endogenous promoter (knock-in)	ETV6-RUNX1 renders mice prone to T-cell malignancy after chemical mutagenesis when expressed in HSCs, but not in early lymphoid progenitors.	2009	[20]
Constitutive expression of ETV6-RUNX1 from the endogenous promoter	No leukemia development was observed without a genetically induced random mutagenesis approach.	2011	[21]
ETV6-RUNX1 expression is driven from a CMV promoter and constitutively activated since fetal HSC stage.	No leukemia development was observed even under low-dose radiation exposure.	2013	[22]
The expression of the fusion protein is restricted to CD19(+) B cells	No leukemia development was observed.	2013	[18]
ETV6-RUNX1 expression is restricted to HS/PCs	Low penetrance of pB-ALL under common infection exposure.	2017	[28]

Given the fact that the *ETV6-RUNX1* fusion gene is only associated to pB-ALL, many genetically engineered mouse models have been designed to express the *ETV6-RUNX1* gene in a committed B-cell (Table 1) [12–17]. These models allow bypassing the limitations and experimental variability of the xenotransplant models, but these mice did not develop hematologic disorder of any kind. Recently, a novel *ETV6-RUNX1* transgenic mouse model with restricted expression of the fusion gene to CD19^+^ B cells was generated. This model showed that *ETV6-RUNX1* induced reactive oxygen species and drove the accumulation of DNA damage in B cells [18]. However, this model did not give rise to leukemia [18]. Even more, when the CD19-*ETV6-RUNX1* model [18] was crossed with a *vav-Bcl2* transgenic model resulted in shortened latency to follicular lymphoma [19], but the double transgenic mice did not develop pB-ALL (Table 1). These attempts to model the childhood ETV6-RUNX1+ pB-ALL by targeting committed B-cells suggest that the cell-of-origin is an undifferentiated hematopoietic precursor cell.

The next-generation *ETV6-RUNX1* mouse models were based on conditional gene targeting of the *Runx1* gene into the endogenous *Etv6* locus of the mouse by which the *ETV6-RUNX1* gene is activated in a temporal- and tissue-specific manner following expression of Cre recombinase [20]. Schindler et al. observed malignancies in the T-lymphoid lineage following ENU mutagenesis when the ETV6-RUNX1 protein is expressed in HSCs [20]. A similar constitutive knock-in approach was employed by van der Weyden et al. [21]. These mice do not develop pB-ALL spontaneously. However, when an insertional mutagenesis screen was performed by intercrossing these mice with those carrying a Sleeping Beauty transposon array, 20% of the offspring developed pB-ALL, but even more often T cell leukemia and myeloid leukemias appeared (Table 1) [21]. Similarly, constitutive activation of *ETV6-RUNX1* in fetal HSCs gave not rise to leukemia development even when the mice were exposed to low-dose radiation [22]. Thus far, no experimental model of translocation t(12;21)(p13;q22) associated leukemia has recapitulated the full disease phenotype of pB-ALL, irrespective of the mode and timing of expression or the source of target cells. The inability to generate spontaneous pB-ALL may reflect the requirement for specific secondary molecular hits, which are not induced efficiently by the nonspecific environmental mutagenesis strategies used.

### The infective theory of *ETV6-RUNX1* leukemia

Infection was the first suggested causal exposure for childhood *ETV6-RUNX1* pB-ALL and remains the strongest candidate [17–22]. However, its role in the conversion of the *ETV6-RUNX1* preleukemic clone into pB-ALL remains unknown. We recently showed that an inherited susceptibility to childhood pB-ALL (*Pax5*+/−) linked to a B-cell deficiency and followed by infection exposure may lead to pB-ALL [23]. However, it is unclear if a somatically acquired gene fusion which occurs in humans prenatally during early steps in haematopoiesis, like in the pB-ALL-associated *ETV6-RUNX1*-translocation, act in a similar manner as an inherited *Pax5*SNP in the germline because *ETV6-RUNX1* [24, 25] and *Pax5* [26, 27] are fundamentally different in terms of inheritance and mode of cellular action. Furthermore, inherited *Pax5* mutations are rare, hence we aimed to prove the theory of exposure to infection in pB-ALL development in a common subtype of pediatric pB-ALL such as *ETV6-RUNX1* positive leukemia. Nevertheless, the lack of genetically engineered human-like *ETV6-RUNX1* pB-ALL models has hampered our better understanding of the pathogenesis of this disease

### Limiting *ETV6-RUNX1* expression to stem cells induces childhood pB-ALL development under common infection exposure

**Figure 2:**
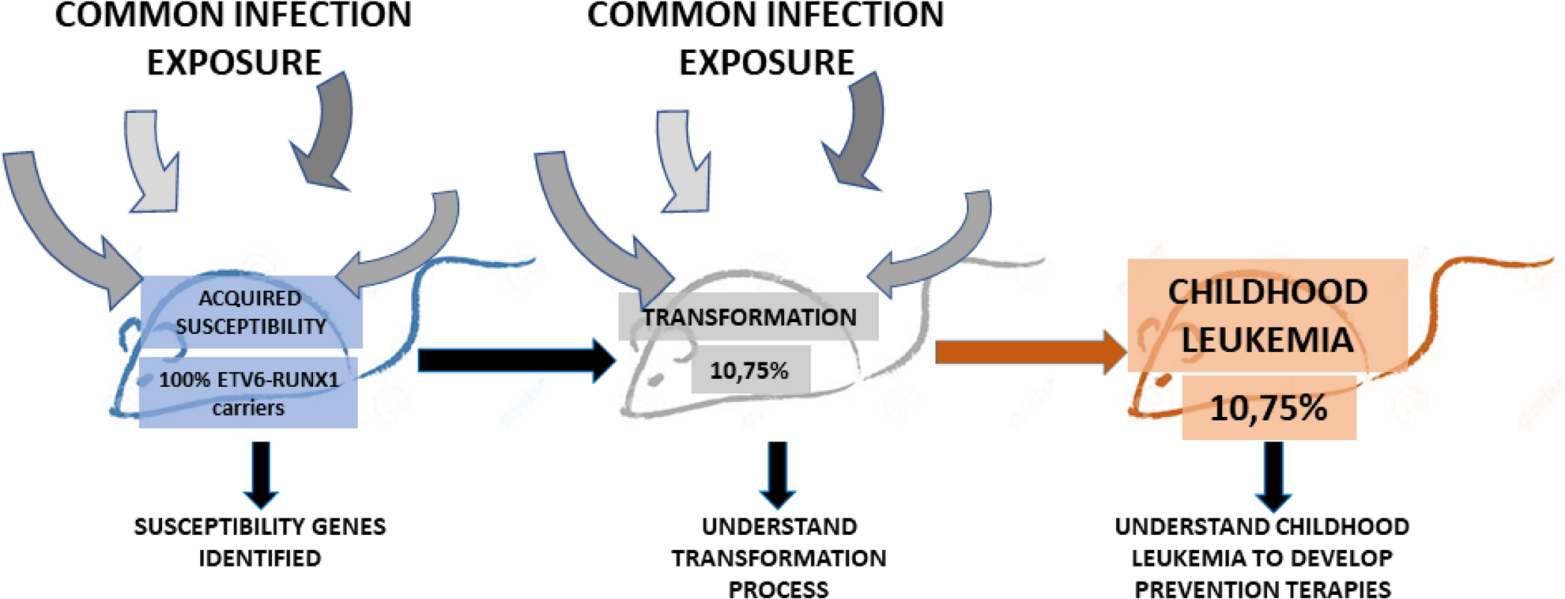
The natural history of ETV6-RUNX1 pB-ALL childhood in the mouse The Sca1-*ETV6-RUNX1* mouse model restricts the oncogen expression to the HS/PCs. The pB-ALL development in this mouse model is driven by the exposure of the mice to common infections and has a low penetrance (10,75%), as in humans. This mouse model is valuable because it allows us to identify susceptible genes and understand the transformation process and hopefully to develop new prevention therapies for pB-ALL childhood leukemia.

Preleukemic clones carrying *ETV6-RUNX1* oncogenic lesions are frequently found in neonatal cord blood [9, 10], where majority of preleukemic carriers do not convert into pB-ALL. These findings suggest that *ETV6-RUNX1* might promote leukemogenesis by creating an aberrant progenitor compartment that is susceptible to malignant transformation through accumulation of additional secondary hits that will be acting as drivers of leukemogenesis. Thus, *ETV6-RUNX1* does not seem to be a dominant oncogene within the natural cellular hematopoietic stem cell compartment where the *ETV6-RUNX1* oncogenic lesion takes place. The existence of this process is, however, difficult to demonstrate using available models of transgenic-driven *ETV6-RUNX1*, because current available models do not mimic the human scenario where majority of preleukemic carriers do not convert into pB-ALL, and the initiating *ETV6-RUNX1* oncogenic lesion that would create the susceptibility is genetically preserved in the cancer-initiating cells, but also through the different pathological cellular intermediates, until the final tumour differentiated cells, and thus making it difficult to ascertain their molecular role at the different stages of tumour biology. Thus, we engineered mice to restrict *ETV6-RUNX1* gene expression to hematopoietic stem/progenitor cells (HS/PCs) in order to unmask this unknown potential mode of action of *ETV6-RUNX1* (Sca1-*ETV6-RUNX1* mice). These Sca1-*ETV6-RUNX1* mice developed exclusively pB-ALL at a low disease penetrance only when they were exposed to common pathogens [28] (Figure 2). This limited expression of ETV6-RUNX1 during early hematopoietic development mimicked human ETV6-RUNX1 preleukemic biology, where *ETV6-RUNX1* expression is not detected in progenitor B-cells of healthy children carrying preleukemic clone [29, 30], and conferred a low risk of developing pB-ALL after exposure to common pathogens corroborating the low incidence in humans and facilitated the identification of genetic basis of the clonal evolution of an ETV6-RUNX1 preleukemic clone to pB-ALL. The infection-driven pB-ALLs that developed in Sca1-*ETV6-RUNX1* mice closely resemble the human disease both in low penetrance and in pathology and genomic lesions (Figure 2). Moreover, these results show that the control of oncogenesis is a function of both the target-cell and the genetic oncogenic alteration(s), being the molecular mechanisms of action of *ETV6-RUNX1* at the HSC level different from those acting at later stages of tumoral cell differentiation [31]. We have shown that the Sca1-*ETV6-RUNX1* model mimicked human *ETV6-RUNX1* preleukemic biology [9, 10] and provided a means to evaluate the potential for oncogenic environments that contribute to pB-ALL development.

### Importance and implications of these findings in childhood pB-ALL development and prevention

**Figure 3:**
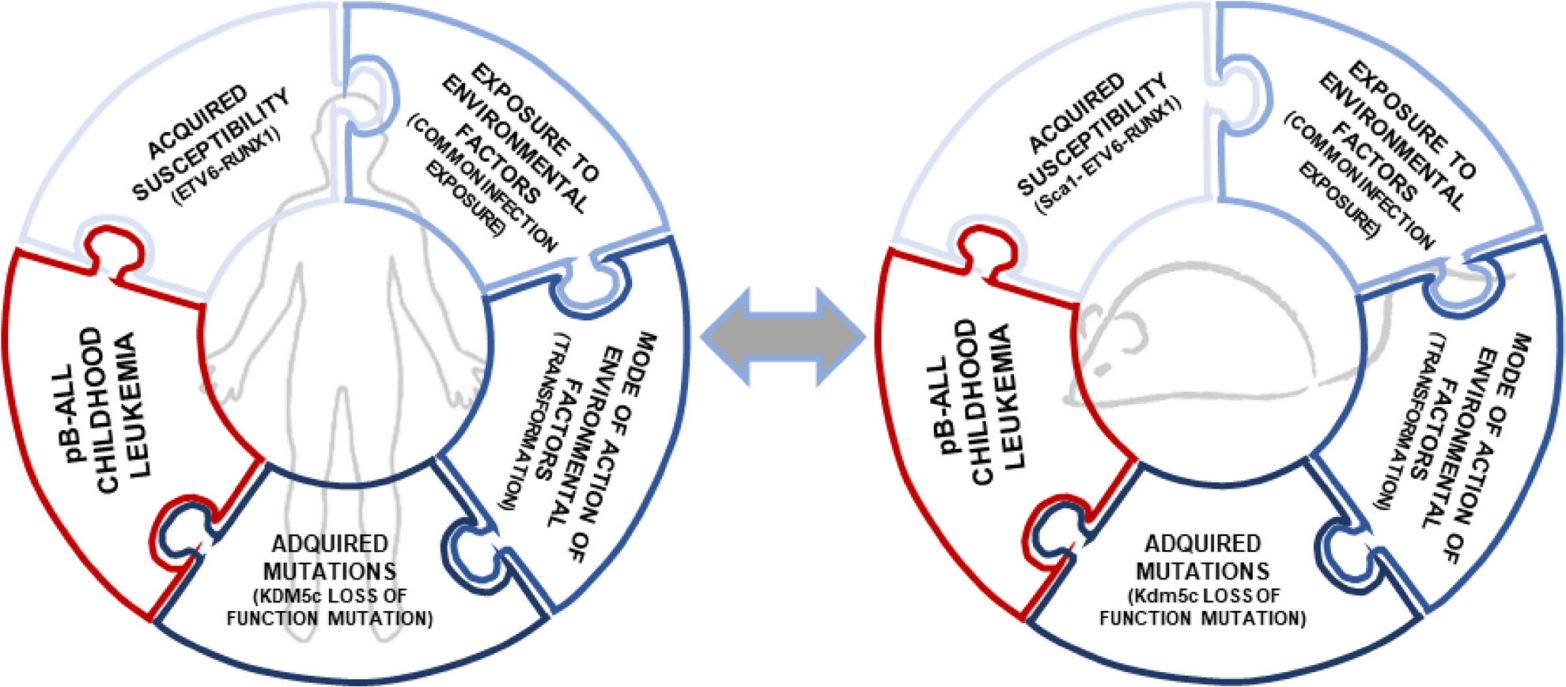
Parallelisms in clonal evolution of ETV6-RUNX1 preleukemic clones to pB-ALL childhoood leukemia in mouse and humans The *ETV6-RUNX1* fusion gene is linked with the most common subtype of childhood leukemia. In humans, only few *ETV6-RUNX1* carriers develop pB-ALL so the essential genetic basis for development of full-blown leukemia remains to be recognized. The infective theory linked to the development of leukemia was postulated 100 years ago and has been validated experimentally in the last two years [23, 28]. Thereby, the clonal evolution of an ETV6-RUNX1 preleukemic clone to pB-ALL is a multistep process in which a preleukemic clone (ETV6-RUNX1+) is transformed only when is exposed to common infections [18]. Similar secondary genetic alterations were found in both human and mice, like the loss of function mutation in *KDM5c* (p.408Y>C mutation in humans and p.804R>^*^ mutation in mice).

This preclinical model of ETV6-RUNX1-associated pB-ALL can be essential tool to prevent the occurrence of this disease in preleukemic carriers for the following reasons: 1) pB-ALL develops *de novo* in the context of the native tissue environment and intact immune system, 2) pB-ALL arises from genetic and/or environmental factors that are relevant for the childhood pB-ALL, 3) the model mimics multistage progression, including preleukemic lesions that have the potential to progress to malignant ones, 4) the murine phenotypes display histological and biological features in common with their human counterpart, 5) murine leukemias present similar molecular pathways that are dysregulated in the human pB-ALL, 6) murine pB-ALL originate in appropriate cells of origin, 7) murine pB-ALL display heterogeneity, as occurs in childhood pB-ALL, and 8) the mice also model genetic diversity, as happens in human populations (Figure 3). So, how could this Sca1-*ETV6-RUNX1* model be fully integrated into childhood pB-ALL prevention? In our view analyses of the Sca1-*ETV6-RUNX1* model offers unique opportunities to interrogate aspects of childhood pB-ALL prevention that are difficult to study in preleukemic carriers such as accessing preleukemia and/or early-stage pB-ALL with known progressive potential. In addition, it will facilitate the discovery of how relevant environmental factors promote leukemogenesis in genetically defined preleukemic contexts. Realizing the benefits of early pB-ALL detection will require improved means of distinguishing, at the earliest possible stages, ‘true’ pB-ALL that require intervention, from the majority of non-malignant ones. In addition, identification of early-stage leukemic lesions (true pB-ALL) as early as possible would probably enable us to prevent or delay progression to clinically relevant disease. In recognition of these challenges, the identification of extrinsic and intrinsic risk factors in healthy preleukemic carriers will allow the goal of minimizing their exposure and thereby reducing leukemia incidence.
